# A haptic laparoscopic trainer based on affine velocity analysis: engineering and preliminary results

**DOI:** 10.1186/s12893-021-01128-z

**Published:** 2021-03-18

**Authors:** Benjamin De Witte, Charles Barnouin, Richard Moreau, Arnaud Lelevé, Xavier Martin, Christian Collet, Nady Hoyek

**Affiliations:** 1 Inter-University Laboratory of Human Movement Science (EA 7424), Univ Lyon, University Claude Bernard Lyon 1, 27-29 Boulevard du 11 Novembre 1918, 69622 Villeurbanne Cedex, France; 2 INSA Lyon, Ampère (UMR5005), Univ Lyon, 25 av. Jean Capelle ouest, 69621 Villeurbanne Cedex, France; 3 Faculty of Medicine, Surgery School, Univ Lyon, University Claude Bernard Lyon 1, 8 Avenue Rockefeller, 69003 Lyon, France; 4 Service d’Urologie et de chirurgie de la Transplantation, Hôpital Édouard Herriot, Hospices Civils de Lyon, 5 Place d’Arsonval, 69003 Lyon, France

**Keywords:** Minimal invasive surgery, Motor skills, Assessment metrics, Affine velocity, Simulator validation

## Abstract

**Background:**

There is a general agreement upon the importance of acquiring laparoscopic skills outside the operation room through simulation-based training. However, high-fidelity simulators are cost-prohibitive and elicit a high cognitive load, while low-fidelity simulators lack effective feedback. This paper describes a low-fidelity simulator bridging the existing gaps with affine velocity as a new assessment variable. Primary validation results are also presented.

**Methods:**

Psycho-motor skills and engineering key features have been considered e.g. haptic feedback and complementary assessment variables. Seventy-seven participants tested the simulator (17 expert surgeons, 12 intermediates, 28 inexperienced interns, and 20 novices). The content validity was tested with a 10-point Likert scale and the discriminative power by comparing the four groups’ performance over two sessions.

**Results:**

Participants rated the simulator positively, from 7.25 to 7.72 out of 10 (mean, 7.57). Experts and intermediates performed faster with fewer errors (collisions) than inexperienced interns and novices. The affine velocity brought additional differentiations, especially between interns and novices.

**Conclusion:**

This affordable haptic simulator makes it possible to learn and train laparoscopic techniques. Self-assessment of basic skills was easily performed with slight additional cost compared to low-fidelity simulators. It could be a good trade-off among the products currently used for surgeons' training.

## Background

Minimally Invasive Surgery (MIS) has brought more comfort for the patients in comparison to open surgery [1–3]. However, MIS also challenged surgeons with an increased need for manual dexterity, depth perception, and movement coordination through a 2D screen displaying the surgical field. Therefore, efficient training of spatial abilities [4], bi-manual coordination [5], and hand–eye coordination [6], are essential for young surgeons [7, 8]. During the past two decades, simulators have been more extensively used in surgeons’ training [9], without the risk of harming patients [10, 11], with health care cost reductions [10], and better efficiency than with simple observation [12]. Two kinds of simulators coexist: low and high fidelity simulators. Low-fidelity ones use simple real instruments and are better suited to novice learners [11] as they are efficient for basic skills acquisition [13] such as clipping, grasping, or cutting. However, they lack fidelity [14] and fail to provide immediate and summative feedback [14–16], which seriously hinders learning [11, 17, 18]. Also, they require a subjective observation by an expert [14, 19], which reduces effective training opportunities for novice surgical students [20]. High-fidelity simulators, such as the LAP Mentor™ (Simbionix Corporation, Cleveland, Ohio, USA) improve the performance of full complex laparoscopic procedures (such as hysterectomy [21]) [9]. Nevertheless, they are very cost-prohibitive and may not be accessible for regular and personal use [19, 22, 23].

Even if low- and high-fidelity simulators may be considered as a continuum in the learning process [13, 17], a gap remains between them: for instance, Yiasemidou et al. [24] showed that the students who autonomously trained on a “take-home” box trainer ($$\approx$$ $500) at home during 6 weeks performed better than the ones who practiced on the High-Fidelity Virtual-Reality-based simulator ($$\approx$$ $70,000). Therefore, a solution mixing low-cost, broad availability, autonomous use, and objective automatic assessment should efficiently fill this gap.

To address the above-mentioned flaws, this study aims to develop and validate a new low-cost ($$\approx$$ $2500) low-fidelity simulator providing: (i) training on basic laparoscopic psycho-motor tasks (requiring spatial and visual-motor capabilities to acquire universal gesture-based skills versus specific surgical ones such as in [25]), (ii) both haptic and summative feedback, and (iii) a new assessment variable (in a similar approach as in [26]) permitting the evaluation of the smoothness of motions and so better expertise discrimination. As shown by Shout et al. [27], before testing the training efficiency of a simulator, scientific validation needs to be processed. According to current standards for educational and psychological testing [28, 29], we present the preliminary validation results (reliability, content, and relation with experimental designs).

## Methods

### Engineering and exercises

**Fig. 1:**
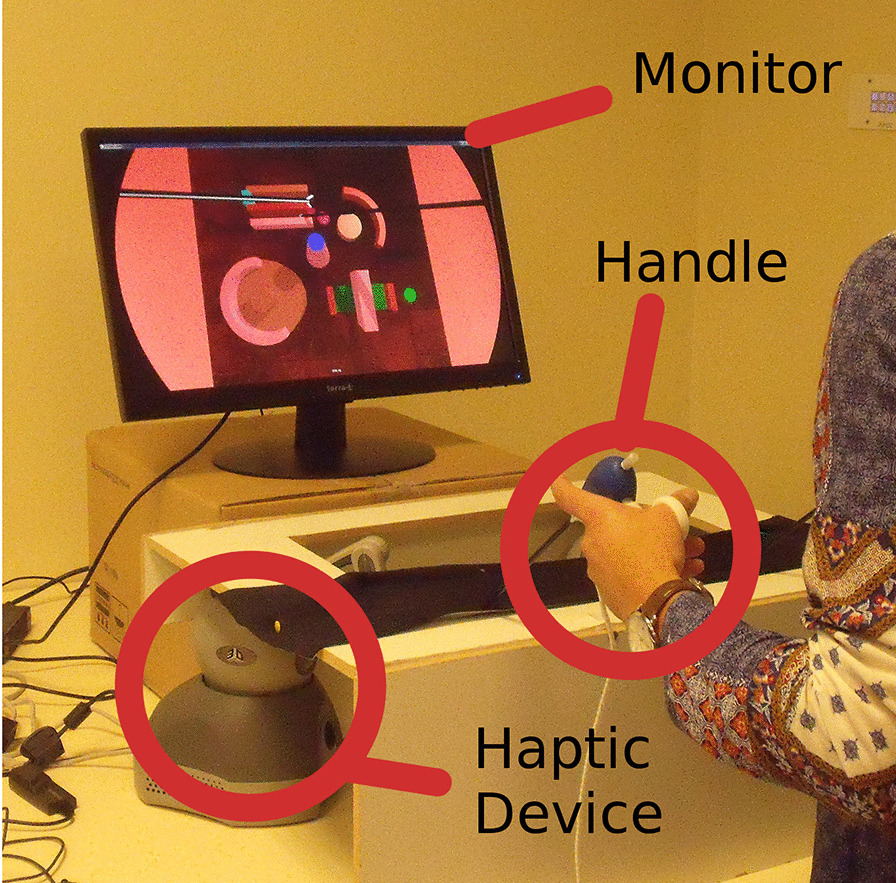
Overview of the simulator. The user manipulates real MIS tools connected to the haptic interface hidden under the cover which mimics the patient’s skin. The trainee monitors his gestures in the virtual environment through the monitor

This simulator was designed by observing surgeons in operation rooms and isolating key-skills with the help of the Fundamentals of Laparoscopic Surgery [30]. Prono-supination, elbow flexion and extension, wrist rotations, and index finger rotations were the basic anatomical movements that were analyzed. The exercises embedded into this simulator require the user to regularly perform these anatomical movements as they consist of navigation tasks using laparoscopic grasping tools while avoiding 3D obstacles displayed on a computer screen. In total, five obstacles are displayed along the trajectory, each one requiring the execution of a specific action (Fig. 1). After the design, the exercises have been submitted to and validated by the Head of Lyon Surgery Department. They combine memory work and skills involving visual-spatial ability [31]. Considering the high cognitive load involved by high-fidelity simulators, which may negatively impact the ability to learn medical gestures [32], we followed the recommendations of Mayer and Moreno [33] and kept the exercises and the virtual environment simple, focusing on the gestures (trajectories and tool synchronization).

To avoid high computing costs, we used open-source software and affordable material [34]. This low-fidelity simulator is a computer-based training system. The hardware includes a computer (Microsoft Windows 7^®^  Intel^®^ Core™ i7-6500 CPU clocked at 2.50GHz, Nvidia^®^ Geforce GTX940M, 8Go RAM), and a standard monitor displaying virtual 3D environment, associated with two real standard laparoscopic surgical instruments, paired each one with a force-feedback device thus simulating tactile and kinesthetic feedback (Geomagic Touch^®^ haptic device, 3D System Inc.). The latter is a 6 degrees-of-freedom device using three electrical motors as actuators, often used in medical simulators [35]. We developed the software with Microsoft Visual Studio^®^ 2015^1^ and we used the open-source Haptic Framework CHAI3D 3.1.1^2^ to make the haptic devices, the 3D virtual world, and the Open-GL 4.4^3^ renderer communicating with each other. We designed all virtual objects with the 3DS Max software 2016.^4^ We finally collected and processed performance data (affine velocity, motion duration, number of collisions) through Matlab^®^ R2015a (Mathworks).^5^

Assessment metrics used in surgical training simulators: completion time, instrument path length, number of collisions (between the laparoscopic tools and their environment), or count of dropped objects, are indirect indicators of laparoscopic proficiency that need to be associated with other objective measures i.e. motion characteristics [15, 36]. A review of existing metrics [26] concludes that available metrics do not allow an objective determination of the detailed expertise of subjects. Moreover, users do not consider these metrics consistent with their performance [37]. Indeed, experiments revealed that experts perform smoother, more accurate, and fine movements compared to novices [38], but usual metrics do not directly evaluate the curvature of instrument motions.

Affine velocity is a metric that takes the geometry of the trajectory into account, as well as its dynamics. In the 2D drawing, humans tend to decrease the instantaneous tangential velocity of their hands while the curvature of the trajectory increases. Correspondingly, the hand velocity increases when the trajectory becomes straight [39]. Furthermore, this relationship conforms to an empirical two-thirds power law [40]. For an MIS-tool 3D trajectory, this property had to be adapted for spatial motion. Some experimental results suggested that the two-thirds power law does not fit 3D motions and that a one-sixth power law is needed [41]. The relation between the Euclidean velocity *v*, the curvature $\kappa$, and the torsion $\tau$ is defined as:

1 $$\begin{aligned} v = v_a \cdot \kappa \cdot \alpha \cdot |\tau | \cdot \beta \end{aligned}$$

where $v_a$ is the affine velocity, $\alpha$ and $\beta$ are two parameters to be determined according to the skill to be studied. For example, in [42], the motion of an obstetrical gesture (typically the installation of forceps during childbirth) was very different from those in MIS. As a result, the parameters used in these previous works could not be used for MIS. Therefore, we had to determine those for MIS, which should fit every motion involved in this hands-on training. This is explained in the next subsection. Affine velocity was utilized to provide a quantitative measure reflecting the quality of the kinematics of users’ trajectories. Affine velocity was used instead of the velocity itself because it provides valuable information about the quality and smoothness of the trajectory. Data from each training session (trajectories, moments, and numbers of collisions, right/left-hand distributions) were collected through CHAI3D into a file and post-processed using Matlab to evaluate the trainees’ performance. In future versions, these assessment algorithms will be integrated into the simulator software.

The first step to tune the affine velocity computation was to get enough data to determine the best $\alpha$ and $\beta$ values for this simulator. We collected an adequate sample of 46 trajectories ranging from novices to experts (see the next section). We independently verified their skill level so that they could serve as references. After the process proposed in [42], we first interpolated the trajectories of both tooltips into cubic splines and computed the values of the Euclidean velocity *v*, the curvature $\kappa$, and the torsion $\tau$ (measuring how much the trajectory twists out of the plane of curvature). Then, we performed a logarithmic linearization of (1):

2 $$\begin{aligned} \log (v) = \log (v_a) + \alpha \cdot \log (\kappa ) + \beta \cdot \log (|\tau |) \end{aligned}$$

We performed multivariate linear regression using a gradient descent algorithm to determine these parameters. The average values of $\alpha$ and $\beta$ should fit the best laparoscopic medical skills to allow a realistic computation of affine velocity. For this medical skill, we found $\alpha$ = − 0.048 and ${\beta =-0.0026}$.

### Validation processes

According to the American Psychological Association guidelines [43] and standards [28], we validated the simulator’s content evidence and discriminative power.

### Content evidence validation and its internal consistency

**Fig. 2:**
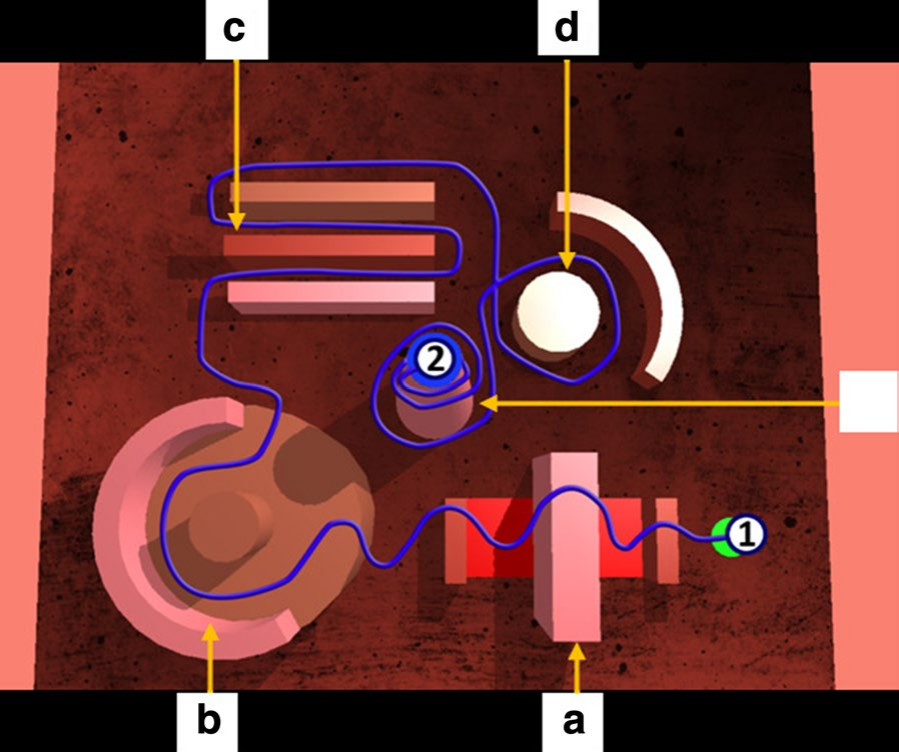
Screenshot of the trajectory to perform in exercise 1 from the starting position (1) to the ending position (2). The trajectory had to be performed without touching the 3D structures. The five obstacle avoidance requested implicitly the following skills: grasping and releasing the green sphere to start, jumping over the central block in A (insertion, withdrawal), taking a curve in B, moving straight between the blocks in C, going round in D, and E, simulating loops necessary for knot tying

**Fig. 3:**
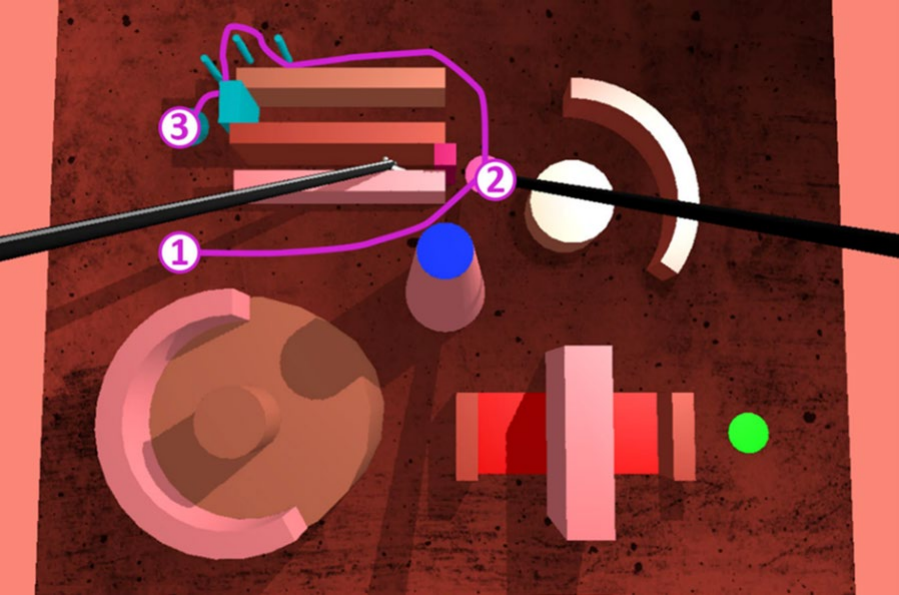
Screenshot of the trajectory to perform in exercise 2 from the starting position (1) to the ending position (3). The participant, using his/her non-dominant hand had to perform a small trajectory (in purple):** a** start from point 1,** b** go to point 2 that is the first virtual button (located under the label 2) he/she had to push to open the pink virtual gate (on the left of label 2),** c** with his/her dominant hand, pass the latter gate,** d** with the non-dominant hand, slalom from point 2 to point 3 between the 3 light blue poles without touching them,** e** once arrived at point 3, the non-dominant hand had to rotate around its axis (using the instrument wheel) to open the light blue gate and enable the dominant hand to go further between the two upper bars (not represented)

We examined if the simulator's items completely represent a basic surgical skill-learning tool. For the first exercise (Fig. 2), participants should replicate the trajectory as fast as possible with their dominant hand only, the non-dominant hand staying motionless while holding the second laparoscopic tool. During the second exercise (Fig. 3), participants had to perform the same trajectory as fast as possible with their dominant hand while the non-dominant hand should simultaneously complete a small trajectory. After completing the two exercises, the participants were invited to globally assess the simulator using a 25-item questionnaire on a 10-point rating scale. Six questions evaluated the general aspect (such as *“How would you rate the height of the simulator regarding a real intervention?”*), five questions were about the didactic components (*How would you rate the quality of the feedback (metrics, score...)?*), four questions were about the perception of reliability (*How would you rate the accuracy of the movements regarding reality?*), seven questions were linked to the training and learning power (*Do you think this simulator could be useful for MIS training?*), and finally, three questions were related to the simulator's utility (*How do you think this simulator improves the use of the non-dominant hand?*). Finally, the internal consistency test ensured the reliability of the responses [44, 45].

### Discriminative validation (relation with experience) and temporal stability

Another approach to validate this simulator was to compare expert and novice performance [46, 47]. The simulator should show that experts perform better than novices. This should indicate that successful performance on the simulator requires surgical expertise. We thus tested here the simulators’ discriminative power by comparing participants’ performance on the two exercises through three variables (time, number of collisions, and affine velocity). Finally, we tested the temporal stability i.e. the reliability of measurements by a test–retest session. Five participants among the experts’ group were randomly selected for this comparison. As recommended, the two testing sessions were separated by at least 1 month without any practice on the simulator [48].

### Participants

Seventy seven participants (mean age = 30.8; female = 26; male = 51) agreed to participate in the study. There were 17 experts (mean age = 41.5; female = 4; male = 13) selected regarding the number of medical procedures they already performed (more than 100 interventions as the main operator). Twelve surgeons of intermediate surgery experience (1 female and 11 males, mean age = 28) were selected in the second group as they had between 5 and 20 interventions as the main operator. Twenty-eight inexperienced interns with basic open surgery experience e.g. knot tying and suturing were included in the third group. They additionally observed laparoscopic interventions without performing any operation. There were 8 females and 20 males in this group (mean age = 25.3). Twenty novices unrelated to medical education or surgical skills were also selected, representing 13 females and 7 males (mean age = 31.5). The latter group did not complete the questionnaire because their opinion was not relevant to the content validation process. Among the expert group, one participant did not complete the questionnaire. This experiment did not require any IRB approval. Verbal consent was provided by every participant. Participants’ demographic and surgical specialty information is summarized in Table 1.

**Table 1:** Participants’ demographic and specialty

Group	General information		Speciality							Experience
	Total number (Male:Female)	Mean age (range)	Paediatric	Gynaecology	Urology	Digestive	Visceral	Orthopaedic	General	
Experts	17 (13:4)	41.5 (62–30)	1	1	8	4	2	1	0	$\ge$ 100 interventions^a^
Intermediates	12 (11 :1)	28 (32–25)	0	0	10	0	2	0	0	5-20 interventions^a^
Inexperienced interns	28 (20 :8)	25.3 (40–23)	0	0	0	0	0	0	28	Weak experience^b^
Novices	20 (7:13)	31.5 (53–18)	0	0	0	0	0	0	0	No experience^c^

^a^As main operator

^b^Never performed any laparoscopic operation but had some basic open surgery experience like knot tying and suturing

^c^Students with no medical education or surgical skills

### Data analysis

Data were analyzed using SPSS 21.0 (SPSS Inc.)^6^. To assess content evidence, we applied a one-factor ANOVA to compare groups with post hoc Tukey correction tests. A level of p $\le$ 0.05 was considered statistically different. Cronbach alpha test measured the internal consistency of the questionnaire with the $\alpha$-value set at 0.7). We used non-parametric tests to compare groups regarding time, number of collisions, and affine velocity (discriminative protocol), as the number of samples was too low for parametric tests. The Kruskal–Wallis test compared the three groups, associated with the Kolmogorov–Smirnov for two-by-two comparisons. The statistical threshold was set at p $\le$ 0.05. Finally, correlation coefficients evaluated the temporal stability of the simulator’s measure.

## Results

### The simulator

We designed and prototyped a training simulator using, as possible, affordable components available on the shelf and standard open-source software technologies. Its cost has been evaluated at around $2500 for a single specimen, including hardware purchases, rapid fabrication, and software development. This cost is compliant with the provision by universities of series of a simulator for free use by students in comparison with the cost of a single high-fidelity simulator (>$50.000).

### Content evidence and its internal consistency

All participants positively rated the simulator whatever the group of inclusion, with mean (±SD) scores being 7.25 ± 0.8, 7.74 ± 0.6, and 7.72 ± 0.7 for the expert, intermediate and inexperienced levels, respectively. ANOVA did not provide any difference among the three groups (p = 0.13, NS). The internal consistency of the content evidence questionnaire revealed a high Cronbach alpha coefficient (0.87).

### Discriminative validation (relation with experience) and temporal stability

#### Exercise 1

*Time* Experts and intermediates completed this exercise faster than inexperienced interns and novices. Mean times were 82.4 s (SD = 22.1 s), 81.5 s (SD = 23.7 s), 122.3 s (SD = 50 s), and 156.8 s (SD = 53 s) for experts, intermediates, interns, and novices respectively. The Kruskal–Wallis test revealed a significant difference among the groups (Chi-square = 32.80, p < 0.001) with a mean rank of 22.41 in the experts, 20.92 in the intermediates, 43.14 in the inexperienced interns, and 58.15 in the novices. Post hoc tests showed significant differences between experts and both inexperienced interns (p = 0.007) and novices (p < 0.001). Intermediates outperformed inexperienced interns (p = 0.01) as well as novices (p < 0.001). Finally, inexperienced interns tend to be faster than the novices (p = 0.06).

*Collisions* Experts and intermediates made fewer collisions than inexperienced interns and novices. The mean number of collisions was 9.4 (SD = 5.8), 8.8 (SD = 4.7), 17.3 (SD = 8.2), and 24.5 (SD = 19.5) for experts, intermediates, interns, and novices, respectively. The Kruskal–Wallis test revealed significant differences among groups, (Chi-square = 22.48, p < 0 .001) with a mean rank of 24.24 in the experts, 22.83 in the intermediates, 46.30 in the inexperienced interns and 51.03 in the novices. Post hoc tests showed significant differences between experts and inexperienced interns (p = 0.004), experts and novices (p < 0.001) and as well as between intermediates and inexperienced interns (p = 0.007); intermediates and novices (p = 0.001).

*Affine velocity* Experts and intermediates outperformed inexperienced interns and novices in the affine velocity variable. Mean affine velocities were 0.023 m/s (SD = 0.0024 m/s), 0.022 m/s (SD = 0.0030 m/s), 0.026 m/s (SD = 0.0055 m/s) and 0.030 m/s (SD = 0.0038 m/s) for experts, intermediates, interns and novices respectively. Kruskal–Wallis test revealed significant differences among groups, (Chi-square = 30.65, p < 0.001) with a mean rank of 25.76 for the experts, 19.08 for the intermediates, 41.86 for the inexperienced interns, and 58.20 for the novices. Post hoc tests showed significant differences between experts and novices (p < 0.001) and between experts and inexperienced interns (p = 0.05). Post hoc tests showed also significant differences between intermediates and inexperienced interns (p = 0.009) as well as between intermediates and novices (p < 0.001). There was also a significant difference between inexperienced interns and novices (p = 0.003).

**Fig. 4:**
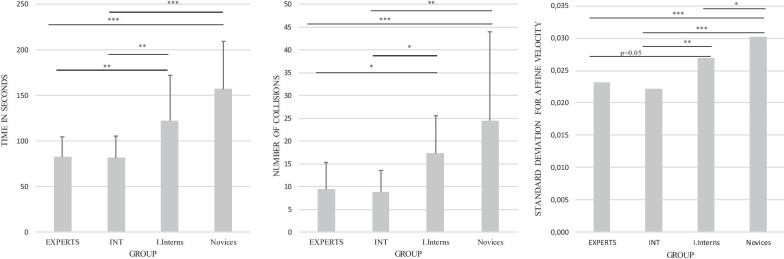
Mean values (SD) for time, collisions, and standard deviation means for affine velocity in exercise 1. For experts, intermediates (INT), inexperienced interns (I. Interns), and novices. *p < 0.05; **p < 0.01; ***p < 0.001

#### Exercise 2

*Time* Experts and intermediates completed the second exercise faster than inexperienced interns and novices. The mean movement time was 109.5 s (SD = 30 s), 107.1 s (SD = 34.1 s), 147.3 s (SD = 41 s), and 188.1 s (SD = 46.6 s) for experts, intermediates, interns, and novices respectively. The Kruskal–Wallis test revealed a significant difference among the groups, (Chi-square = 34.09, p < 0.001) with a mean rank of 21.97 in the experts, 21.88 in the intermediates, 42.09 in the inexperienced interns, and 59.43 in the novices. Post hoc tests showed significant differences between experts and inexperienced interns (p = 0.01); experts and novices (p < 0.001) but also between intermediates and inexperienced interns (p = 0.02) as well as between intermediates and novices (p < 0.001). Movement time was also different between inexperienced interns and novices (p = 0.02).

*Collisions* Experts and intermediates made fewer collisions than inexperienced interns and novices. The mean number of collisions was 17.7 (SD = 10.3), 19.6 (SD = 5.9), 23.3 (SD = 13), and 37.5 (SD = 20.1) in experts, intermediates, interns, and novices respectively. The Kruskal–Wallis test revealed significant differences among groups, (Chi-square = 14.703, p = 0.002) with a mean rank of 27.21 in the experts, 33.88 in the intermediates, 37.52 in the inexperienced interns, and 54.18 in the novices. Post hoc tests showed significant differences between experts and novices (p = 0.002) and between intermediates and novices (p = 0.03). There was also a significant difference between inexperienced interns and novices (p = 0.03)

*Affine velocity* Experts and intermediates outperformed inexperienced interns and novices. Mean affine velocity were 0.028 m/s (SD = 0.0042 m/s), 0.029 m/s (SD = 0.0055 m/s), 0.032 m/s (SD = 0.0056 m/s) and 0.033 m/s (SD = 0.0051 m/s) in experts, intermediates, interns, and novices, respectively. The Kruskal–Wallis test revealed significant differences among groups, (Chi-squared = 10.41, p = 0.015) with a mean rank of 26.88 in the experts, 31.83 in the intermediates, 42.89 in the inexperienced interns and 48.15 in the novices. Post hoc tests showed significant differences between experts and novices (p = 0.04) and tend to be different between experts and inexperienced interns (p = 0.06).

**Fig. 5:**
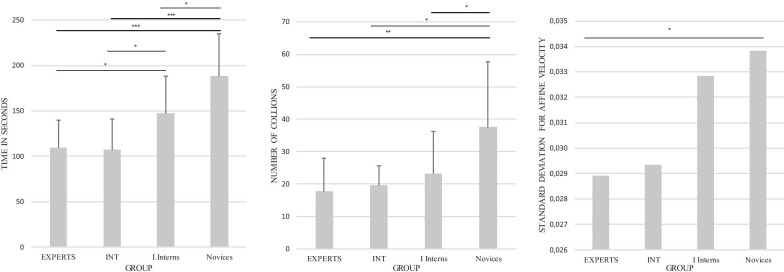
Mean values (SD) for time, collisions, and standard deviation means for affine velocity in exercise 2. For experts, intermediates (INT), inexperienced interns (I. Interns), and novices. *p < 0.05; **p < 0.01; ***p < 0.001

Finally, regarding the temporal stability of the simulator’s measure, we found a correlation for the number of collisions and for the time variables between the two sessions (r = 0.89; p = 0.04).

## Discussion

This study demonstrated the reliability and content evidence of this low-fidelity simulator in laparoscopic motor skills learning. The difference among groups’ performance accounts for the discriminative power of the simulator. The users uniformly rated the way the simulator exhibits actual motor abilities required by real surgery. Surgeons’ global agreement about the usefulness of the simulator for surgical curricula is attested through a large number of participants, where three levels of practice were represented (from inexperienced interns to expert surgeons, with an intermediate level). The outcome scores are in line with the outcomes from other studies dealing with the validation of computer-based systems [49]. For instance, the MIST VR (MenticeMedical Simulation, Gothenburg, Sweden) was rated by an average score of 7 in a comparable study [49]. Since this validation process, several studies used the MIST VR to train basic laparoscopic skills, thereby demonstrating the relevance of the simulator in surgery training [47, 50]. Taken together, the surgeons validated positively how laparoscopic skills are brought into play and assessed in the simulator. Regarding its discriminative power, both exercises exhibited a slightly different pattern of results. In general, we observed that experts and intermediates outperformed inexperienced interns and novices according to the three dependent variables we selected in both exercises, i.e. movement time duration, number of collisions, and affine velocity (Figs. 4 and 5). This confirmed that the simulator discriminated among four expertise levels although to a lesser extent between inexperienced interns and novices. The simulator thus highlighted the main abilities needed by MIS. When looking closer to the results of exercise 1, experts and intermediates generally performed at the same level. This outcome is consistent as both groups had real experience and this first exercise did not require advanced laparoscopic skills. The same argument can explain the discrepancy between these groups and the two others, which did not master laparoscopic motor skills, even basic ones. This is a positive outcome as our purpose was to engineer a low-fidelity simulator for the training of basic surgical skills, thereby more fitted for beginners [13]. In the second exercise, the pattern of results was quite similar to that of the first exercise. However, when looking closer at data (Fig. 5), only the novice group is systematically outperformed by the other groups (except for affine velocity where only the experts outperformed the novices). To better analyze and interpret these results, we should consider that the task was bi-manual. Surgery skills required bi-manual coordination [51] and this ability is essential in laparoscopic surgery [7, 52] and open surgery [5]. Thus, prior open surgical experience helped the experts, intermediates, and inexperienced interns to overcome exercise 2, while the novices did not benefit from previous experience. As a consequence, a laparoscopic simulator should include training for bi-manual tasks.

Motor skills learning is a rapid process when training begins. Then, it slows down to tend towards an asymptote, in reference to a classic learning curve. This is probably why it was difficult to clearly distinguish performance between expert and average surgeons [53]. They were categorized on the basis of professional experience duration and this is not a discriminative factor when testing basic skills. In relation to sporting skills [54], we can keep this hypothesis to be further tested. Expertise is a long process integrating speed, accuracy, and economy of resources, together constituting efficiency. As early as 1995, DesCôteaux and Leclère [55] considered that *“visuospatial perceptual skills (the ability to represent mentally the physical environment and the movement to be performed) are the major determinants of surgical technical performance”*. From this view point, our hypothesis would be tested by integrating other variables associated with a surgical experience, specifically by making the simulator testing specific skills, regarding surgical specialties.

Testing the simulator through one single trial may be seen as a limitation. However, running several trials would include the risk of habituation, i.e. starting a fast learning stage, as observed by Dayan and Cohen [56]. This can potentially affect the outcomes, particularly in the novices and inexperienced interns. Despite only one trial was performed, thus guaranteeing that no learning process could occur, performance between inexperienced interns and novices was often comparable and the difference was weak when reaching significance (see variable “time” during exercise 1 in “Results” section). This shows that medical experience with the mastery of specific skills is not generalized to surgery. Therefore, this should prompt to form only one group of novices in future experiences [56].

Taken together, the results nevertheless suggest that the three variables were relevant. These should be analyzed in a complementary way to provide an objective overview of the trainee’s skills. This confirms the existing literature, stating that time and collision are indirect indicators of laparoscopic proficiency, needed to be associated with motion characteristics, provided by the affine velocity [15, 36].

The evolution of the simulator could incorporate new skills such as suturing and knot tying with the aim to test more advanced techniques. As previously proposed, introducing specific surgical techniques remains an open option. This would require additional software development, however without hardware changes. This would also require coding new exercises in C++, based on the Chai3D library. From our own experience, we estimate that the conception of a single new exercise testing a new skill would require around one person.month of work.

## Conclusion

The outcomes showed that integrating the affine velocity metrics is a reliable tool according to both inquiry results and statistical tests. Also, the simulator exhibits innovative devices providing easy testing through several games and autonomic assessing of the main laparoscopic basic skills. It provides objective experience discrimination which is lacking in low-fidelity simulators [14, 15, 19], for a low additional cost compared to high-fidelity simulators [22]. It thus proposes a pragmatic trade-off between functionalities and cost. However, this probably needs to be confirmed by investigating the implementation of this simulator in an early training stage along with considering the impact of haptic feedback on learning, and the validation of the discriminative power of affine velocity on more difficult exercises.

## Data Availability

The datasets used and/or analyzed during the current study are available from the corresponding author on a reasonable request.

## Abbreviations

ANOVA: Analysis of variance
IRB: Institutional Review Board
MIS: Minimally Invasive Surgery
NS: Non significant
SD: Standard deviation

## Footnotes

1 see https://www.microsoft.com/france/visual-studio/.

2 see http://www.chai3d.org/.

3 see https://www.opengl.org/.

4 see http://www.autodesk.fr/products/3ds-max/overview.

5 see https://fr.mathworks.com.

6 See http://www.ibm.com/software/analytics/spss/products/statistics/index.html
